# Effect of virtual reality exercise on interventions for patients with Alzheimer’s disease: A systematic review

**DOI:** 10.3389/fpsyt.2022.1062162

**Published:** 2022-11-09

**Authors:** Yali Yi, Yuanyan Hu, Mengxin Cui, Cheng Wang, Jibing Wang

**Affiliations:** ^1^School of Mathematics and Statistics, Southwest University, Chongqing, China; ^2^School of Mathematics and Statistics, Yulin Normal University, Yulin, China; ^3^Qingdao Mental Health Center, Qingdao University, Qingdao, China; ^4^Department of Sports, Northwestern Polytechnical University, Xi’an, China; ^5^International College of Football, Tongji University, Shanghai, China

**Keywords:** virtual reality, Alzheimer’s disease, systematic review, exercise, internation

## Abstract

Virtual reality (VR) interventions are increasingly being used in rehabilitating and treating patients with neurological disorders. This study aimed to explore the effects of VR exercise interventions for patients with Alzheimer’s disease (AD). A systematic review of the published literature on randomized controlled trials of VR technology applied to patients with AD was conducted using the preferred reporting entry for systematic reviews and Meta-analysis guidelines. Descriptive analyses were performed to assess the quality of the studies in terms of the characteristics of the included studies, samples, diagnoses, types of VR technologies, subjective and objective levels of immersion, and quality of studies. Eight studies were included, including a pooled sample of 362 patients with AD. A systematic review showed that most studies focused on patients with AD’s cognitive and physical functions. The main finding was that VR interventions could help improve cognitive and physical balance in patients with AD. However, future studies should emphasize design and use well-accepted assessment tools to validate the effects of VR interventions further.

## Introduction

Alzheimer’s disease (AD) is a progressive brain disease and is currently one of the most common causes of dementia worldwide (1). The incidence of AD increases after age 65 and is more common in women (2, 3). Numerous risk factors influence the development of AD, including age, gender, education, socioeconomic status, smoking, alcohol consumption, obesity, physical activity, hypertension, diabetes, hypercholesterolemia, head trauma, sleep disorders, and depression (4). Currently, Food and Drug Administration-approved AD therapies provide symptom relief benefits for some individuals but neither prevent nor improve the disease (5). Once clinicians make a clinical diagnosis of AD in older adults, adults often want to know what they can do to slow cognitive decline with non-pharmacologic treatments (6). Additionally, people also want to know what they can do to delay the onset or reduce their risk of developing AD (7).

Virtual reality (VR) technology has been widely used in rehabilitating older adults with medical conditions to enhance their ability to perform activities of daily living in the last years (8, 9). VR technology has also been reported to be used for people with mild cognitive impairment (MCI) (10), people at high risk of cognitive decline (11); memory rehabilitation patients with noncommunicable diseases (NCDs) (12); patients with NCDs due to traumatic brain injury (13), stroke patients (14, 15), and patients with severe NCDs due to AD (16, 17). VR is an emerging technology which creates a three-dimensional environments digitally, allows people to interact, provides sensory input, and tracks change (18). Several commercial VR gaming systems, such as Dance Revise, Microsoft Xbox Kinect, Nintendo Switch, and Wii Sports provide simulated and realistic exercise experiences (19). Real-time feedback, movement reflection, and rewards in a three-dimensional (3D) world for older patients are provided to encourage motivation to adhere to exercise and willingness to continue exercising (20).

Virtual reality exercise training has become a popular method of treating individuals with mental illness. VR environments add additional spatial features to enhance the cognitive benefits of exercise. Several studies have also shown that VR may improve psychological functioning in patients with cognitive impairment (21, 22). In the treatment of depressive symptoms, older adults facing visual and auditory stimuli in VR may experience an increase in energy, enjoyment of exercise, tranquility, and a decrease in negative feelings. Also, VR exercise supports older adults’ decision-making, emotional regulation, and interaction skills to promote psychological, emotional, and social benefits (23, 24).

Virtual reality technology is an innovative approach to rehabilitation that minimizes the adverse effects of NCDs on individuals, families, and society and has been used in healthcare and rehabilitation (25–28). Current research findings on VA technology being used to improve cognitive function are inconsistent (29). The effects of VR technology on cognitive function in MCI patients were summarized by Wu et al. (10) but they did not offer sufficient data. A scoping review on VR therapies’ impact on people with MCI or dementia was unable to clarify how VR affects people with MCI (30). Several recent original studies of VR interventions in cognitive function recently supplemented the field with new evidence (31–34). Another typical intervention population for VR cognitive training includes people with MCI (9, 35). However, current evidence is less reported on interventions for individuals with AD, and a comprehensive understanding of the effects of VR motor interventions on individuals with AD is needed (36). That is, the overall description of the use of VR in AD disease rehabilitation remains uncertain (21). This review study aims to analyze experimental studies of VR and synthesize its effects on intervention outcomes in AD patients, including cognitive function, daily activities, and physical functioning.

## Methods

### Data sources and search strategies

A systematic review was conducted using different combinations of keywords (“virtual reality,” “video games,” “interactive games,” and “VR”) and (“Alzheimer,” “Alzheimer’s disease,” and “AD”) with these terms in the databases Web of Science and PubMed/MEDLINE from inception to December 2021. Two reviewers and the corresponding author collaborated to develop the search strategy, and the two reviewers independently completed the search and cross-validated it. Endnote X7 software was used for literature management.

### Study selection

The inclusion criteria for the study literature were (1) VR interventions for adults with AD; (2) Measurement of cognitive indicators or physical function indicators pre- and post-VR interventions; (3) Interventions that included the use of an immersive or non-immersive VR technology targeting cognitive or physical function in patients with AD; (4) A controlled experimental study, and (5) Studies are provided in English. We excluded studies based on the following characteristics: (1) Case studies; (2) Feasibility studies; (3) non-AD patient-focused studies; (4) Study protocols and reviews; (5) Expert letters, editorials, notes, and book chapters; and (6) Conference abstracts and papers.

### Data extraction

First, titles and abstracts were screened using eligibility criteria by two independent reviewers. Studies that met the criteria for inclusion or were uncertain were kept for full-text scanning. Disagreements on unclear studies were resolved in consultation with the corresponding authors. Second, two independent reviewers assembled the following details from each study: publication details (including author, year, and title), study design (objectives, methods, and setting), participants (including sample size, mean age, and gender), VR intervention (including name of VR, technical details, subjective and objective immersion levels, number and frequency of VR workouts, duration of each VR workout, and total mean duration of VR), outcome measures, results, user acceptance, adverse effects, and summary of intervention effects.

### Levels of virtual reality immersion

The term “immersion” refers to a method for describing how much human participants’ senses may be fooled into believing something is genuine by computer displays. Generally, the level of the immersion technology is evaluated using five criteria: (1) Inclusiveness indicates the extent to which physical reality is rejected; (2) Extensiveness indicates the range of sensory modalities to which it is adapted; (3) Surrounding shows how wide-ranging this VA is as opposed to being restricted to a narrow field; (4) Vivid conveys fidelity, the resolution, and a range of energies (e.g., visual and color resolution) of the simulation within a given channel. The degree of vividness is related to the richness of the display, the content of the information, the resolution, and the quality. These aspects of immersion are related to the display of information; and (5) Matching requires a match between the participant’s proprioceptive feedback about body movement and the information generated on display (37, 38).

### Quality assessment

Two reviewers (YY and MC) independently assessed the quality of the inclusion literature using the PEDro scale (38). The scale consists of 11 items, with the first item not scored and a maximum score of 10. Studies scoring ≥6 was considered high quality, while those scoring below six were considered low quality (39). Two reviewers resolved disagreements in scoring in consultation with the corresponding authors (YH and JL).

### Statistical analysis

The literature was critically analyzed based on (1) study characteristics, sample, type of VR technology, and intervention duration and frequency; (2) VR immersion level; and (3) quality assessment of the study and risk of bias. The applicability of the meta-analysis was considered concerning the available information.

## Results

### Study selection

**FIGURE 1:**
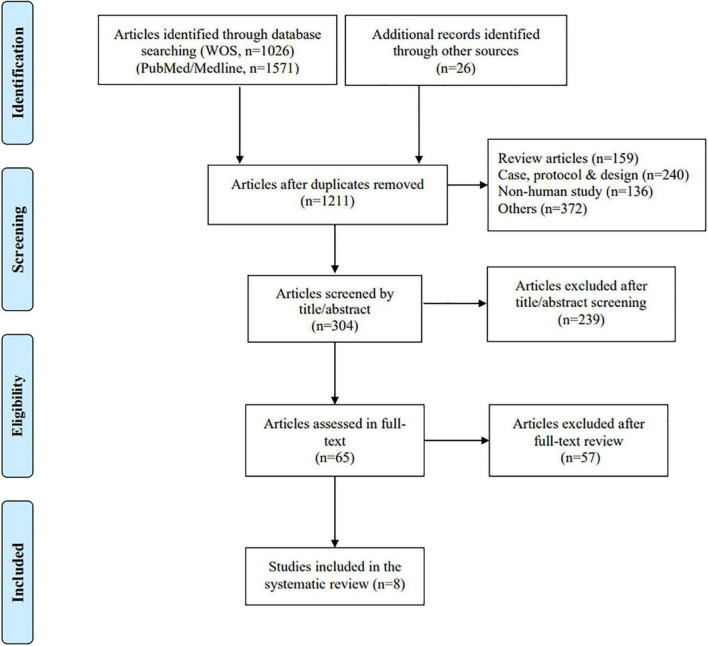
PRISMA flowchart of included and excluded studies.

Eight of the 2,023 studies were included in the systematic review, and the total sample of AD patients participating in these studies was 362 (Figure 1). A meta-analysis is not feasible considering the variability of the study results.

### Study characteristics

Table 1 lists each study sample’s characteristics, study design, intervention type, measurement outcomes, and intervention effects. The study sample was 59.1% female, and the experimental design was a non-randomized controlled trial in only one study (29). The type of exercise intervention was a combination of VR and cognitive or motor exercise; the outcome measures were mainly cognitive, memory, and executive function, with some studies involving physical function. All included studies showed positive changes, with AD patients showing improvements or enhancements in cognitive function, executive function, memory, and body balance. Overall, the different VR interventions showed positive outcomes or feasibility of implementing the intervention.

**TABLE 1:** Characteristics of the inclusion studies.

ID	References	Age (years)	Sample size (all/female)	Design	Intervention exercise	Measurements	Intervention effect
1	Anderson-Hanley et al. (40)	EG: 75.9 ± 9.9 CG: 81.6 ± 6.2	79/62	Cluster RCT	CyberCycle training	Cognitive function physical health exercise behaviors executive function	Compared to traditional exercisers, older adults who Cybercycled had better cognitive function.
2	Karssemeijer et al. (41)	79.2 ± 6.9	115/53	RCT	Exergame training aerobic training	FICSIT-4 PASE score Katz Index EFIP TOPICS	EG showed a trend toward higher adherence compared to AG. Comparing the EG to the CG, a significant decrease in the EFIP was observed.
3	Oliveira et al. (44)	EG: 82.6 ± 5.42 CG: 84.14 ± 6.3	17/12	Pilot RCT	Non-immersive VR	Executive function global cognition functionality depression	Executive function did not show improvement. A significant effect was in the global cognition.
4	Padala et al. (45)	EG: 72.1 ± 5.3 CG: 73.9 ± 7.1	30/11	Prospective RCT	Yoga strength training aerobics balance games	Balance fear of falling quality of life cognitive function functional state	Compared to the walking group in BBS, the Wii-Fit group showed a noticeably larger improvement.
5	Park et al. (29)	83.81 ± 2.66	13/11	Trail	Episodic memory training	BBS ABC; FES 3MS; MMSE ADL; IADL QOL-AD	The difference in BBS from baseline between the Wii-Fit group and the walking group was noticeably different.
6	Serino et al. (43)	EG: 86.6 ± 6.13 CG: 88.7 ± 3.59	20/17	RCT	VR-Based training	Cognitive domains	Following VR-based training for AD patients, there was a noticeable increase in long-term spatial memory.
7	Ugur and Sertel (4)	EG: 73.75 ± 5.16 CG: 73.13 ± 3.54	32/9	RCT	Balance and aerobic exercises with VR	Cognition balance	The balance and walking speed were enhanced by VR applications.
8	Werner et al. (42)	82.7 ± 6.2	56/39	RCT	Exergaming	Motor-functional cognition motor-cognitive dual-task performance	A significant improvement in all Physiomat^®^ tasks already after 3 weeks.

CG, control group; EG, experimental group; VR, virtual reality; RCT, randomized controlled trials; 3MS, Modified Mini-Menta; ABC, Activities Specific Balance Scale; ADL, activities of daily living; BBS, Berg Balance Scale; EFIP, evaluative frailty index for physical activity; FES, Falls Efficacy Scale; FICSIT-4, frailty and injuries cooperative studies of intervention techniques subtest4; IADL, Instrumental Activities of Daily Living; MMSE, Mini-Mental State Exam; QOL-AD, Quality of Life-AD; TOPICS, The Older Persons and Informal Caregivers Survey Minimum Dataset.

### Virtual reality technology and levels of immersion

Table 2 shows the titles of the different VR applications, VR immersion levels, VR usage settings, VR user experiences, and their adverse feedback reported in studies. Regarding the description of VR immersion levels, as provided by the studies, 50% of the technologies were described as semi-immersive (4, 40–42), 25% as immersive (29, 43), and 25% as non-immersive (44, 45). The mean duration of a single intervention was 30 min (SD = 12.8, range = 10–50) in the eight studies; the mean number of interventions was 23 (SD = 13.2, range = 10–40); and the mean duration of total intervention activities was 758 min (SD = 628.7, range = 200–1,800). Interestingly, no adverse events were reported in 62.5% of the included studies (4, 29, 43, 44). Of the adverse events reported, one study had four adverse events, but they were not considered relevant to the study (41); another study reported in detail four participants dropping out midway through the intervention due to severe medical time and seven participants dropping out after the intervention due to lack of motivation (*n* = 4), the injury falls (*n* = 2), and death (*n* = 1) (42). One additional study provided 13 adverse events in an attachment, including motor impairment, nausea and disorientation, neck pain, and dizziness (39).

**TABLE 2:** Virtual reality (VR) levels of immersion, characteristics of the VR programs, and user experience.

References	Name of VR application	Subjective level of immersion	Number of VR sessions and frequency	Length of each VR session	User acceptance	Adverse effects
Anderson-Hanley et al. (40)	CyberCycle	Semi-immersive	5 times*8 weeks	45 min	Interactive experience	13 adverse events
Karssemeijer et al. (41)	Stationary bike connected to a video screen	Semi-immersive	3 times *12 weeks	30–50 mins	A fun way of exercising	No occurrence of serious adverse events
Oliveira et al. (44)	VR computerized program	Non-immersive	2 times *6 weeks	45 min	Not specified	Not specified
Padala et al. (45)	Wii-Fit interactive	Non-immersive	5 times *8 weeks	30 min	Fun and interactive	4 adverse events Not related to study.
Park et al. (29)	E-Prime software	Immersive	2 times *8 weeks	20 min	Not specified	Not specified
Serino et al. (43)	NeuroVirtual 3D	Immersive	3 times *3–4 weeks	20 min	Not specified	Not specified
Ugur and Sertel (4)	Nintendo Wii	Semi-immersive	2 times *6 weeks	30 min	Not specified	Not specified
Werner et al. (42)	Exergame-based balance training system (Physiomat^®^)	Semi-immersive	2 times *10 weeks	10 min	The increasing difficulty of the Physiomat^®^ tasks	Lack of motivation (*n* = 4), injurious falls (*n* = 2), and death (*n* = 1)

Table 3 shows the objective level of immersion based on the FIVE criteria developed by Slater et al. (37). Many studies (62.5%) met the criteria for moderate immersion (40, 42–45), with the remainder being 12.5% high immersion (4), 12.5% low immersion experiences (41), and 12.5% information unavailable for immersion (29).

**TABLE 3:** Immersion levels of virtual reality (VR) technologies.

References	Inclusiveness	Extensiveness	Surrounding	Vividness	Matching	Total score	Numerical score
Anderson-Hanley et al. (40)	Low	Moderate	High	Moderate	Low	Moderate	1.8
Karssemeijer et al.(41)	Low	Moderate	Moderate	Low	Low	Low	1.4
Oliveira et al. (44)	Low	Moderate	Moderate	Moderate	Moderate	Moderate	1.8
Padala et al. (45)	Low	Moderate	Moderate	Moderate	High	Moderate	2.0
Park et al. (29)	Moderate	Moderate	Moderate	Info unavailable	Moderate	Info unavailable	–
Serino et al. (43)	Low	Moderate	High	Moderate	Moderate	Moderate	2.0
Ugur and Sertel (4)	Low	Moderate	Moderate	High	High	High	2.2
Werner et al. (45)	Low	Moderate	Moderate	Moderate	Low	Moderate	1.6

### Quality assessment

The PEDro scale was used to assess the methodological quality of the eight studies included in the systematic review. The PEDro score was below six in only one study (43) and ≥6 in the other seven studies, indicating good quality of the included studies. However, we also discovered fewer studies in the PEDro quality evaluation on participant and therapist blinding. Only three studies blindfolded the participants, and only one research blinded the therapists. Details of the raw records are shown in Table 4.

**TABLE 4:** Studies quality assessment.

ID	References	Eligibility and source	Random allocation	Concealed allocation	Groups similar at baseline	Participants blinding	Therapist blinding	Assessor blinding	<15% dropouts	Intension-to- treat analysis	Between-group difference reported	Point estimate and variability reported	Total
1	Anderson-Hanley et al. (40)	Y	Y	Y	Y	N	N	Y	N	Y	Y	N	6/10
2	Karssemeijer et al. (41)	Y	Y	Y	Y	N	N	N	Y	Y	Y	Y	7/10
3	Oliveira et al. (44)	Y	Y	Y	Y	Y	N	N	Y	N	Y	Y	7/10
4	Padala et al. (45)	Y	Y	Y	Y	Y	N	N	Y	N	Y	Y	7/10
5	Park et al. (29)	Y	Y	N	Y	N	N	N	Y	Y	Y	Y	6/10
6	Serino et al. (43)	Y	Y	N	Y	N	N	N	Y	N	Y	Y	5/10
7	Ugur and Sertel (4)	Y	Y	Y	Y	N	N	N	Y	Y	Y	Y	7/10
8	Werner et al. (45)	Y	Y	Y	Y	Y	Y	Y	N	N	Y	Y	8/10

## Discussion

The present study aimed to investigate the impact of VA on the effectiveness of interventions for patients with AD. Descriptive analyses were conducted to assess the quality of the study characteristics, study sample, diagnosis, type of VA technology, level of subjective and objective immersion, and quality assessment of eight studies with a total sample of 362 AD patients. Our two independent evaluators extracted information separately and simultaneously to improve the accuracy of the screening process for inclusion in the studies.

The main finding was that VR interventions for patients with AD showed improvements in cognition, memory, executive function, and body balance at various levels. VR applied to interventions for patients with AD had significant effects. This systematic review’s findings supports the use of VR therapies for cognitive rehabilitation and physical function enhancement.

The subjective level of immersion reported for most VR technologies was described as semi-immersive. The average number of VR exercises was 23, with each lasting approximately 30 min, 2–3 times per week. Thus, the frequency and effectiveness of VR interventions appear to be acceptable as part of an innovative initiative. Most studies did not report adverse events. The adverse events addressed in some of the studies were also not caused by the studies. Only in one study were two falls and one death reported in detail at the end of the participant’s intervention (41). Thus, future use of VR interventions for patients with AD will need to be monitored for adverse effects, but these effects do not constitute a reason to exclude the use of VR.

Most studies met the objective criteria for moderate immersion. It is common knowledge that greater levels of immersion may improve the user experience and significantly impact the sensation of presence. However, in VR applications for AD patients, promoting a high level of immersion that corresponds to the experience of feeling “present” in the real world is critical to the patient’s response to the intervention. In the available literature, we cannot yet determine whether fully immersive VR technologies are better than moderate or low immersion VR technologies (46–48).

Although the overall quality of the study was good, adverse effects must be kept ensuring that study participants are blinded to the interventions they receive. This will ensure that evidence is presented favorably in the analyses that lead to the main findings (49).

## Conclusion

We systematically reviewed the literature on the use of VR technology for rehabilitating cognition and its physical function in patients with AD. VR interventions help improve cognition, memory, executive function, and physical balance. VR interventions for the rehabilitation of AD patients are an innovative approach. Despite the possibility that this systematic review does not lend itself to a meta-analysis, the findings indicate that VR therapies are useful and can potentially improve patients with AD’s physical function and cognitive rehabilitation.

Based on the findings of this review, we recommend the following for future research. (1) Studies focus on the experimental design of VR interventions, especially the design of randomized controlled trials blinded to the method. (2) Compare different levels of immersion, explore the differences between full immersion and moderate and low immersion, and develop optimal immersion application strategies. (3) Systematically assess user perceptions and adverse effects. (4) Use widely used outcome metrics to evaluate cross-cultural effectiveness. (5) Use well-promoted VR programs to promote comparability of intervention effects.

## Data availability statement

The original contributions presented in this study are included in the article/supplementary material, further inquiries can be directed to the corresponding authors.

## Author contributions

YY and MC: data collection. YY, YH, and CW: data analysis, conception, and design. YY, YH, and JW: research design, writing the manuscript, and revision. All authors contributed to the article and approved the submitted version.
